# Ellagic acid, a phenolic compound, exerts anti-angiogenesis effects via VEGFR-2 signaling pathway in breast cancer

**DOI:** 10.1007/s10549-012-1977-9

**Published:** 2012-02-21

**Authors:** Neng Wang, Zhi-Yu Wang, Sui-Lin Mo, Tjing Yung Loo, Dong-Mei Wang, Hai-Bin Luo, De-Po Yang, Yu-Ling Chen, Jian-Gang Shen, Jian-Ping Chen

**Affiliations:** 1 School of Chinese Medicine, Li Ka Shing Faculty of Medicine, The University of Hong Kong, Estates Building, 10 Sassoon Road, Hong Kong, China; 2 The First Affiliated Hospital, Sun Yat-Sen University, Guangzhou, 510080 China; 3 School of Pharmaceutical Sciences, Sun Yat-Sen University, Guangzhou, 510080 China; 4 Faculty of Pharmacy, The University of Sydney, Sydney, NSW 2006 Australia

**Keywords:** Ellagic acid, Anti-angiogenesis, VEGF/VEGFR2, Molecular docking, Breast cancer

## Abstract

Anti-angiogenesis targeting VEGFR-2 has been considered as an important strategy for cancer therapy. Ellagic acid is a naturally existing polyphenol widely found in fruits and vegetables. It was reported that ellagic acid interfered with some angiogenesis-dependent pathologies. Yet the mechanisms involved were not fully understood. Thus, we analyzed its anti-angiogenesis effects and mechanisms on human breast cancer utilizing in-vitro and in-vivo methodologies. The in-silico analysis was also carried out to further analyze the structure-based interaction between ellagic acid and VEGFR-2. We found that ellagic acid significantly inhibited a series of VEGF-induced angiogenesis processes including proliferation, migration, and tube formation of endothelial cells. Besides, it directly inhibited VEGFR-2 tyrosine kinase activity and its downstream signaling pathways including MAPK and PI3K/Akt in endothelial cells. Ellagic acid also obviously inhibited neo-vessel formation in chick chorioallantoic membrane and sprouts formation of chicken aorta. Breast cancer xenografts study also revealed that ellagic acid significantly inhibited MDA-MB-231 cancer growth and P-VEGFR2 expression. Molecular docking simulation indicated that ellagic acid could form hydrogen bonds and aromatic interactions within the ATP-binding region of the VEGFR-2 kinase unit. Taken together, ellagic acid could exert anti-angiogenesis effects via VEGFR-2 signaling pathway in breast cancer.

## Introduction

In recent decades, breast cancer has attracted global concern in recent decades. The global burden of breast cancer has engineered oncologists to develop novel strategies for breast cancer prevention and treatment [1, 2]. It has been recognized that angiogenesis is one of the essential hallmarks of cancer, typically breast cancer. To sustain growth, tumors require sufficient nutrients, oxygen, and certain effective ways to evacuate waste. All the aforementioned needs can be addressed by angiogenesis, the process that consistently forms novel blood vessels into tumor masses from existing endothelium-lined vessels. Besides, extensive laboratory evidences supported that tumoral angiogenesis can be detected throughout the onset, growth, and metastasis in breast cancer [3, 4]. Thus, the anti-angiogenesis therapy has become one of the most important modalities in breast cancer treatment.

Angiogenesis process is orchestrated by a balance between pro- and anti-angiogenic factors. During breast cancer progression, angiogenesis occurs when the activity of stimulators exceeds that of inhibitors. After vessel invasion into breast tumor masses, there are at least six different angiogenesis-associated growth factors secreted, among which vascular endothelial growth factor (VEGF) is the most potent angiogenesis stimulator [5, 6]. The specific action of the VEGF on the endothelial cells is mainly mediated by two types of receptor tyrosine kinases (RTKs), namely VEGFR-1 (Flt-1) and VEGFR-2 (KDR in human/Flk-1 in mice) with high affinities. Of the two receptors, VEGFR-2 plays a more important role in mediating the mitogenesis and permeability of endothelial cells. Activation of VEGFR-2 contributes to phosphorylation of multiple downstream signals including ERK, JNK, and AKT that subsequently promote proliferation, migration, and tube formation of endothelial cells. Considering anti-angiogenesis therapy is to target endothelial cells that support tumor growth rather than cancer cells themselves, VEGFR-2 has become an important therapeutic target for cancer anti-angiogenesis therapy [7–10].

VEGFR-2 belongs to the most aggressive RTKs. Agents now targeting RTKs in oncology can be roughly divided into three categories—antibodies targeting RTK ligands (i.e., targeting VEGF), antibodies targeting receptors themselves (i.e., targeting VEGFR-2), and small molecular inhibitors targeting VEGFR-2 kinase domain [11]. The recombinant of the three-dimensional crystal structure of VEGFR-2 kinase domain provided a basis for structure-based design of small molecular inhibitors. Structurally, VEGFR-2 consists of 1,356 amino acids in humans, and can be separated into three domains: the extracellular VEGF-binding domain consisting of seven immunoglobulin-like segments, the transmembrane domain, and the intracellular catalytic domain possessing tyrosine kinase activity. Upon specifically binding to VEGF, VEGFR-2 would undergo dimerization within the extracellular domain and autophosphorylation within the intracellular catalytic domain by consuming ATP, accompanied by following activation of downstream signaling cascades that stimulate angiogenesis. The ATP-binding site and less conserved surrounding sites are thus especially important for anti-VEGFR2 agent design [12, 13].

Various orally active small molecular inhibitors of VEGFR-2 are now in clinical trials including sunitinib, vandetanib, and sorafenib [14]. Disappointedly, long-duration treatment with these agents might be accompanied by distinct adverse effects such as hemorrhage, hypertensive crisis, and gastrointestinal perforation [15]. Therefore, there has been renewed interest in natural inhibitors that could block VEGFR activation. Many natural phenolic compounds or their specific derivatives are found possessing potent anti-cancer properties [16–18]. Among them, ellagic acid (4,4′,5,5′6, 6′-hexahydroxydiphenic acid 2,6,2′6′-dilactone) is a representative small molecular polyphenol widely found in fruits and vegetables. Extensive studies have reported that ellagic acid exerts potent antioxidant effects either by directly acting as an antioxidant or by activating cellular antioxidant enzyme systems [19, 20]. In addition, ellagic acid was also found to be effective in anti-carcinogenesis through inhibiting tumor cell proliferation, inducing apoptosis, breaking DNA binding to carcinogens, and most importantly, disturbing angiogenesis processes required for tumor growth [21]. However, the mechanisms involved were not thoroughly elucidated.

In this study, the effects of ellagic acid on inhibiting breast cancer angiogenesis were validated in vitro and in vivo. Mechanistic study further indicated that ellagic acid could significantly inhibit VEGFR-2 kinase activity and block its signaling pathway both in vitro and in vivo. Meanwhile, the structure-based interaction between ellagic acid and VEGFR-2 was found to be stable conformation based on in-silico analysis which revealed that hydrogen bond and aromatic interactions were formed.

## Materials and methods

### Cell culture and drug preparation

The human breast cancer cell line MDA-MB-231 was purchased from the American Type Culture Collection (ATCC), and maintained in L-15 medium supplemented with 10% FBS, penicillin (100 U/ml), and streptomycin (100 μg/ml). Human umbilical vein endothelial cells (HUVEC) were cultivated in gelatinized culture plates in M199 medium supplemented with 15% FBS, 1% PS, 50 μg/ml endothelial cell growth supplement (ECGS, BD Bioscience) and 100 μg/ml heparin at 37°C in a humidified atmosphere containing 5% CO_2_. Ellagic acid (≥98%, Sigma-Aldrich, St. Louis, MO) was dissolved in dimethyl sulfoxide (DMSO, final concentration is 0.1%) was diluted in 1× PBS to prepare required concentrations. In each treatment, the cells were treated with vehicle or with various concentrations of ellagic acid in the presence of 20 ng/ml human VEGF (Pepro Tech, Rocky Hill, NJ).

### Cell counting assay

HUVECs were seeded in a 6-well plate with a density of 2 × 10^4^ cells per well at 37°C and 5% CO_2_ for overnight attachment. Then the cultivated medium was discarded and replaced with fresh serum-free medium with or without drug involvement in the presence of VEGF. At the time intervals of 12, 24, and 48 h, cells were harvested for counting in the presence of trypan blue. All samples were assayed in triplicate to generate proliferation curves as described.

### BrdU incorporation assay

DNA synthesis was determined by BrdU labeling assay using Cell Proliferation ELISA, BrdU (colorimetric) kit. In brief, 3,000 HUVECs per well were seeded in a gelatin-coated for overnight attachment. Then the cultivated medium was replaced with serum-free medium supplemented with 20 ng/ml VEGF as well as different concentrations of ellagic acid in a final volume of 100 μl/well. After 24 h, cells were labeled with BrdU (2 h, 37°C), incubated with FixDenat solution (30 min, 20°C), and re-incubated with Anti-BrdU POD (90 min, 20°C). The sample absorbance was finally detected in an ELISA reader at 450 nm.

### Lactate dehydrogenase (LDH) toxicity assay

The LDH released into cell cultures is an index of cytotoxicity and evaluation of the permeability of cell membrane. HUVEC were seeded in 96-well plate at a density of 3,000 cells per well. After incubation with various concentrations of ellagic acid for 24 h, cell supernatants were collected and analyzed for LDH activity using LDH cytotoxicity assay kit from Caymen Company. The absorbance of formed formazan was read at 490 nm on a microplate reader.

### Wound-healing assay

HUVEC cells were seeded in a 6-well plate at a density of 5 × 10^5^/well. After the cells had reached 90% confluency, a scrape was made in the middle of the plate by a 10 μl tip with the gap widths between 150 and 200 μm. After scrapping, the cells were washed with PBS twice, and then incubated with fresh medium containing VEGF with or without ellagic acid (10 μM). Migration ability was evaluated by measuring the gap widths narrowed down by HUVECs movement at different time intervals of 0, 12, and 24 h. Images were then taken with a microscope video system, and three places of each gap were measured and averaged.

### Invasive assay

Transwell model (8-μm pore size) was applied to perform the invasive assay. The upper side of transwell was coated with Matrigel, while the lower compartment was coated with type I Collagen. Then the pre-coated transwell was placed into a well of 6-well plate containing 1 ml complete M199 medium. HUVECs (2 × 10^5^ cells per well) were suspended in 1 ml medium containing various concentrations of ellagic acid and VEGF, and then added to the upper chamber. After incubation at 37°C for 24 h, the non-migrated cells in the upper surface of membrane were removed with a PBS-soaked cotton swab. Then the membranes were fixed with 4% PFA for 30 min. Cells that migrated to the lower side of the membranes were stained with HE method. Photographs were taken by a microscope video system. The number of cells that crossed the membrane in the treated group was compared with that in the control group.

### Tube formation assay

The tube formation assay was performed using 12-well plate coated with 100 μl Matrigel basement membrane matrix (BD Bioscience, Bedford, MA) per well and polymerized at 37° for 30 min. HUVECs suspended in M199 medium containing 2% FBS were plated on the Matrigel at a density of 2 × 10^5 ^cells/well. Different concentrations of ellagic acid (2.5, 5, and 10 μM) were then added together with VEGF (20 ng/ml). After 8 h, The Matrigel-induced morphological changes of HUVECs and their formed tube networks were observed under a microscope and photographed at a five-fold magnification.

### Kinase activity detection

In vitro VEGFR-2 tyrosine kinase activity was assayed using an enzyme-linked immunosorbent assay kit (Boehringer Mannheim, SA). In brief, ellagic acid was incubated with VEGFR-2 (Upstate) in assay buffer containing Mg^2+^ and ATP in 96-well plate coated with a poly-Glu-Tyr substrate. Phosphorylated tyrosine was then detected by sequential incubation with a mouse IgG anti-phosphotyrosine antibody and an HRP-linked sheep anti-mouse immunoglobulin antibody. Color was developed with an HRP chromogenic substrate and quantified by an ELISA reader at wavelength 450 nm. The results were expressed as percent kinase activity.

### Western blotting

In brief, cell lysates (30 μg) were separated by 8% SDS-PAGE and transferred to polyvinylidene difluoride membranes. Membranes were then incubated with primary antibodies including phosphorylated and/or total VEGFR-2, ERK, AKT, JNK, eNOS, and β-actin (Cell Signaling Technology, Danvers, MA). After overnight incubation at 4°C, membranes were washed with TBST three times and then incubated with secondary antibodies at room temperature for 2 h. Immunoreactive bands were then visualized by the enhanced chemiluminescence (ECL) detection system (GE healthcare).

### Gelatin zymography

Supernatants from a HUVEC culture system with or without ellagic acid treatment were collected for MMPs activity analysis by sodium dodecyl sulfate-polyacrylamide gel electrophoresis under non-reducing conditions. One milligram per milliliter of gelatin was prepolymerized on a 10% polyacrylamide gel as a substrate. Electrophoresis was carried out at 4°C. The gel was washed with washing buffer (50 mM Tris–HCl, pH 7.5, 100 mM NaCl, and 2.5% Triton X-100), followed by incubation with a buffer (50 mM Tris–HCl, pH 7.5, 150 mM NaCl, 10 mM CaCl_2_, 0.02% NaN_3_, and 1 μM ZnCl_2_) at 37°C for 16 h and visualized with Coomassie Blue R-250.

### Measurement of reactive oxygen species

2′7′-Dichlorofluorescein diacetate (DCFH-DA, Sigma, St. Louis, MO) was used to measure ROS formation. After exposed to different concentrations of ellagic acid for 24 h, endothelial cells were then incubated in DCFH-DA containing medium (final concentration: 10 μM) at 37°C for 20 min. Cells were washed with PBS three times to remove DCFH-DA that not entered in cells. The fluorescence was visualized immediately at wave lengths of 485 nm for excitation and 530 nm for emission by inverted fluorescence microscope. Total green fluorescence intensities of each well were quantified using image analysis software.

### Chick aortic ring models

In brief, the aortic arches were dissected from 12-day-old chick embryos, cut into rings and embedded into Matrigel in a 6-well plant. After overnight incubation at 37°C, rings were added with serum-free medium containing ellagic acid in the presence of VEGF. Sprouts were formed within 48–72 h. Images were photographed at 5× magnification of a Zeiss inverted microscope. The extent of sprouts formation from chick aortic ring was quantified using image-pro software.

### Chick embryo chorioallantoic membranes (CAM) models

5-Day-old fertilized chicken eggs were incubated at 37°C in 60% humidity for additional 48 h. On day 3, a small hole in the shell concealing the air sac was made with a hypodermic needle. By candling, a second hole was made on the broad side of the egg directly over the avascular portion of the embryonic membrane. A false air sac was created beneath the second hole by applying negative pressure through the first hole, causing the CAM to separate from the shell. A window of about 1 cm^2^ was cut into the shell over the dropped CAM using a small grinding wheel. VEGF (20 ng/ml) was used as a standard proangiogenic agent. Sterile disks of filter paper (Whatman International) were pretreated with 3 mg/ml cortisone acetate and air dried under sterile conditions. The disks were suspended in PBS containing different concentrations of ellagic acid and VEGF, and then placed on the growing CAMs. After 3 days, the tissue directly beneath each disk was reselected from the control and the treated CAM samples. Each tissue sample was washed three times with PBS and digital images were collected. The images were then analyzed by Image-J software. Six regions were randomly selected and the number of vessel branch points contained in a circular region was counted. The resulting angiogenesis index was expressed as mean ± SD of the branch points for each CAM sample. The experiments were repeated three times.

### Xenograft models and immunohistochemistry detections

Use of animal has been approved by the Committee on the Use of Live Animal in the Teaching and Research of the University of Hong Kong. 3 × 106 Human breast cancer MDA-MB-231 cells were implanted into the mammary glands of female, 4-week-old BALB/c nude mice to build breast cancer xenograft. Mice with appropriate size of tumors were divided randomly into three groups including control group, ellagic acid low-dosage group (50 mg/kg/day), and ellagic acid high-dosage group (100 mg/kg/day). The mice were treated with ellagic daily by intraperitoneal administration. Tumor volume and mice body weight were measured every 3 days. Tumor volume was detected by caliper measurements and determined by formula: ½ * width^2^ * length. After sacrificing mice on day 25, tumors and normal tissues will be harvested for molecular assessment. Specifically, tumors and normal tissues were fixed in 10% paraformaldehyde/PBS, dehydrated in 70% ethanol, embedded in 4% paraffin at 4°C, and then sectioned (4 μm). Deparaffinized tumor sections were stained with specific antibodies including CD31, P-VEGFR2, P-AKT, and P-JNK (Cell Signaling) Detection was done with avidin–biotin–HRP complex (Thermo scientific, Fremont, CA) and diaminobenzidine as chromogen. Nuclei were counterstained with hematoxylin. Besides, deparaffinized normal tissue sections including heart, liver, spleen, lung, and kidney will be stained with hematoxylin and eosin (H&E) for toxicological analysis.

### Molecular docking

The LigandFit algorithm in Discovery Studio 2.1 with the CHARMm engine was applied in our molecular docking study. Chemoffice2002 (CambridgeSoft, Cambridge, MA) were used for the drawing of chemical structures. The LigandFit algorithm employed the strategy including initial generation of ligand orientations within VEGFR-2 catalytic domain followed by molecular dynamics (MD)-based simulated annealing, and final refinement by energy minimization. The crystal structure of VEGFR-2 was obtained from Protein Data Bank (http://www.rcsb.org/pdb/) with the PDB ID of 1Y6B. The water molecules in VEGFR-2 were removed. For the docking purpose, the ATP site within VEGFR-2 was defined as the ligand-binding site following the template of 1Y6B, and ellagic acid was docked into VEGFR-2 with proper parameter setting. Specifically, starting from the initial configuration, 100 different orientations of ellagic acid were randomly generated and docked into the ATP pocket of VEGFR-2. All the 100 docking poses were presented simultaneously for the analysis of the interactions between ellagic acid and VEGFR-2. A docking without any output pose was considered as a failure.

## Results

### Ellagic acid inhibited proliferation of HUVECs induced by VEGF

**Fig. 1:**
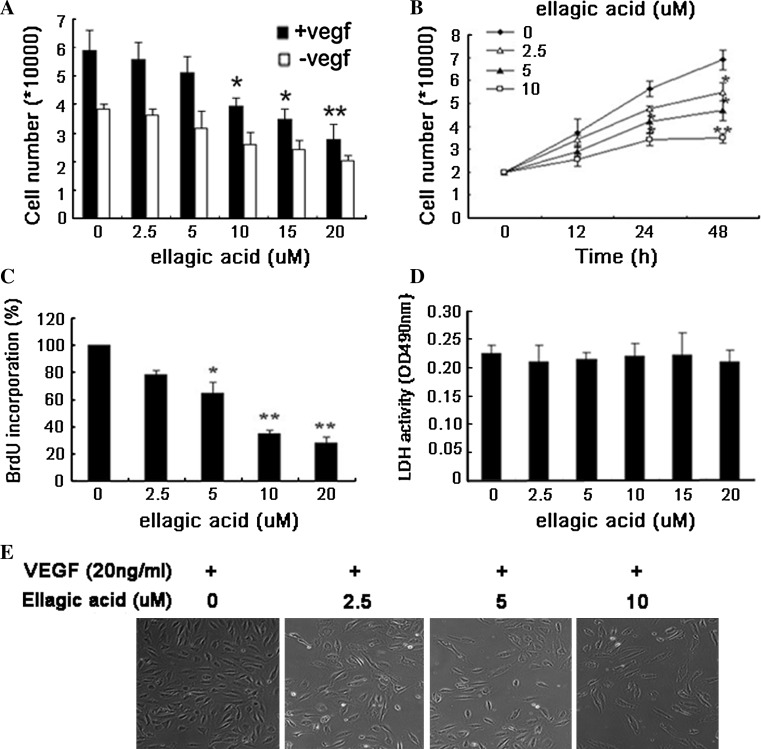
Ellagic acid inhibited proliferation of HUVECs induced by VEGF. **a** After the treatment with ellagic acid at different concentrations ranging from 2.5 to 20 μM for 48 h in the presence or absence of VEGF, cell number was counted. The results showed that the proliferation of HUVECs stimulated by VEGF was significantly decreased in a dose-dependant manner, while ellagic acid had little inhibitory effects on HUVECs that were not stimulated by VEGF (values represent means ± SD, *n* = 6, *P < 0.05, **P < 0.01, versus untreated control). **b** Time-course study indicated that ellagic acid could markedly inhibit endothelial cell proliferation in a time-dependant manner (values represent means ± SD, *n* = 6, *P < 0.05, **P < 0.01, versus untreated control). **c** Effects of ellagic acid on DNA synthesis were examined by BrdU cell proliferation enzyme-linked immunosorbent assay. The results suggested that ellagic acid could obviously inhibit DNA synthesis of HUVECs in a dose-dependent manner (values represent means ± SD, *n* = 6, *P < 0.05, **P < 0.01, versus untreated control). **d** The supernatants of HUVECs after ellagic acid treatment were collected for cytotoxicity examination by LDH cytotoxicity assay kit. The results showed that ellagic acid administration did not result in LDH release from endothelial cells, indicating that ellagic acid posed little cytotoxicity effects upon HUVECs (values represent means ± SD, *n* = 6). **e** VEGF-induced HUVECs were treated with ellagic acid at different concentrations for 48 h, and then photographed by at 100-fold magnification for the detection of HUVECs morphological changes. Little abnormal morphological changes of HUVECs were observed after 48-h exposure to ellagic acid. The results further demonstrated that there was no significant toxicity of ellagic acid at any tested concentration in the cellular level

To examine the anti-angiogenic effects of ellagic acid in vitro, proliferation of VEGF-induced HUVECs was detected first [22]. Previous studies reported that cell number decreased while MTT formazan formation increased in the presence of polyphenol-associated compound (kaempferol) [23]. Therefore, cell counting assay was conducted rather than MTT assay in this study. As shown in Fig. 1a, the proliferation of endothelial cells stimulated by VEGF was markedly decreased after ellagic acid treatment ranging from 2.5 to 20 μM. Besides, HUVECs not containing VEGF showed obscure changes in cell proliferation, indicating extracellular VEGF acted as a strong attractant for endothelial cells proliferation. Then we treated endothelial cells with ellagic acid at different time intervals of 12, 24, and 48 h. The results presented in Fig. 1b showed that ellagic acid significantly suppressed endothelial cell proliferation in a time course. As detected by BrdU incorporation assay (Fig. 1c), DNA synthesis of HUVECs was also significantly inhibited by ellagic acid in a dose-dependant manner. To validate whether or not ellagic acid would result in toxicity effects on HUVECs, LDH cytotoxicity assay was carried out. As shown in Fig. 1d, ellagic acid brought little toxic effects on HUVECs. Meanwhile, there were little abnormal morphological changes of endothelial cells after ellagic acid administration for 48 h (Fig. 1e). We came to the conclusion that ellagic acid at non-cytotoxic doses could significantly inhibit proliferation of VEGF-stimulated endothelial cells.

### Ellagic acid suppressed VEGF-induced migration and tube formation of HUVECs

**Fig. 2:**
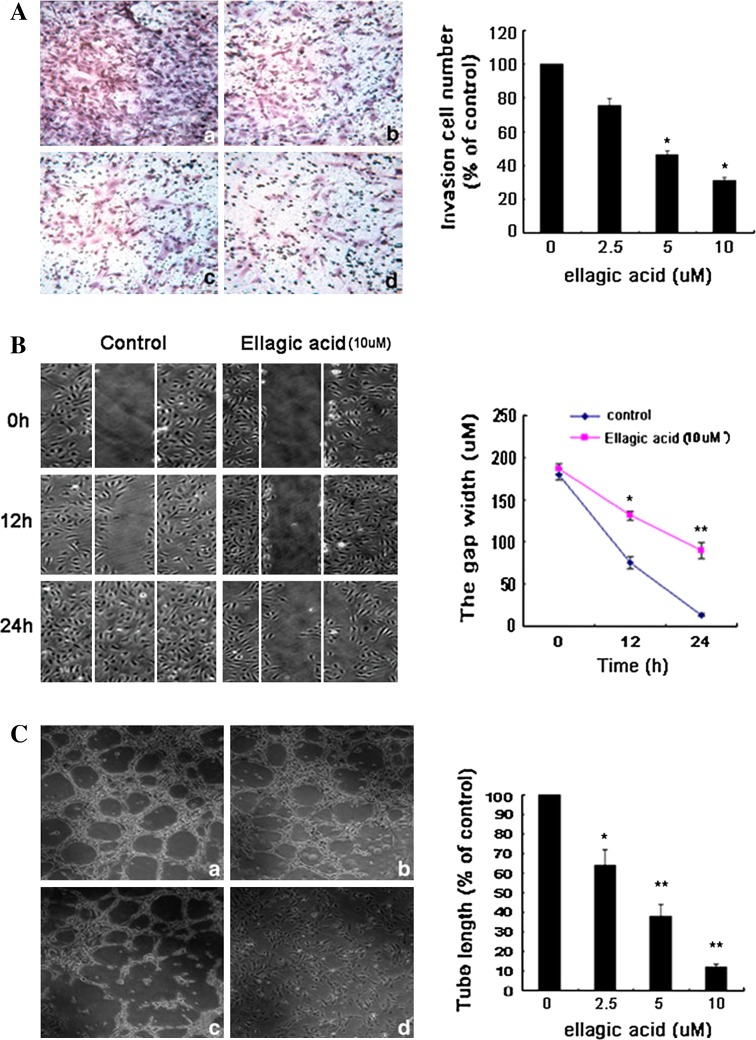
Ellagic acid suppressed VEGF-induced invasion, migration, and tube formation of HUVECs. **a** HUVECs at a density of 2 × 10^5^ cells/ml were planted into a 6-well transwell (8-μm pore size) subsequently added with different concentrations of ellagic acid and VEGF. After 24 h incubation, the cells at the bottom of the membrane were fixed and analyzed. It was showed that ellagic acid decreased the number of invasive cells in a dose-dependent manner (*a* 0 μM, *b* 2.5 μM, *c* 5 μM, *d* 10 μM; data are presented as means ± SD, *n* = 6, *P < 0.05 versus untreated control). **b** A total of 5 × 10^5^ HUVECs per well were seeded in a 6-well plate for wound-healing assay. The results showed that ellagic acid significantly inhibit the migration of HUVECs in the presence of VEGF stimulation (data are presented as means ± SD, *n* = 6, *P < 0.05, **P < 0.01 versus untreated control). **c** HUVECs were seeded at a density of 2 × 10^5^ cells per well using a 12-well plate which was pre-coated with Matrigel. Varying concentrations of ellagic acid (2.5, 5, and 10 μM) were added together with VEGF (20 ng/ml) for additional 8 h incubation. It was observed that ellagic acid could dose dependently suppress the capillary lengths of VEGF-stimulated endothelial cells (*a* 0 μM, *b* 2.5 μM, *c* 5 μM, *d* 10 μM; values represent means ± SD, *n* = 6, *P < 0.05, **P < 0.01 versus untreated control)

Then, we studied the potential of ellagic acid in blocking the migration and tube formation abilities of HUVECs [24]. First, transwell assay demonstrated that the invasive cells to the lower chamber were significantly decreased with the increasing concentration of ellagic acid (Fig. 2A), indicating that the migration ability of HUVECs was dose dependently suppressed by ellagic acid. Meanwhile, the influence of ellagic acid on migration ability of endothelial cells was also assessed by wound-healing assay. As shown in Fig. 2B, the gap width of control group narrowed more obviously than that of ellagic acid-treated group from 0 to 24 h, reflecting that ellagic acid could time dependently weaken the migration capability of endothelial cells. Then the effects of ellagic acid on tube-formation ability of HUVECs were tested on Matrigel. In Fig. 2C, HUVECs showed well-formed tubular structure in the absence of ellagic acid. Capillaries were gradually abrogated due to increasing ellagic acid concentrations. Almost 80% destruction of tube network was observed when HUVECs were incubated with ellagic acid at 10 μM. Taken together, ellagic acid suppressed the migratory process and tube formation activities of endothelial cells in the presence of VEGF.

### Ellagic acid blocked angiogenesis in chick-associated models

**Fig. 3:**
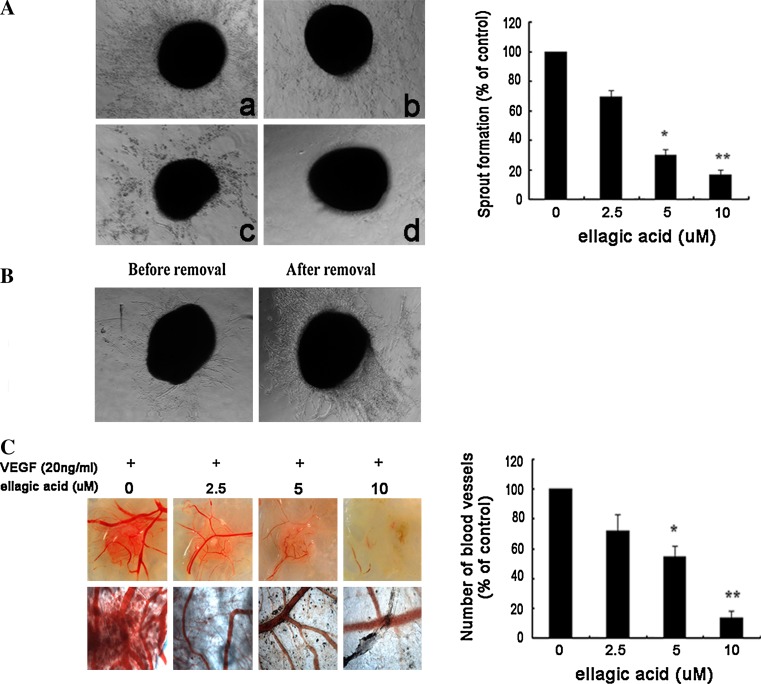
Ellagic acid blocked neo-angiogenesis in chick-associated models. **a** Ellagic acid dose dependently suppressed sprout formation on the organotypic model of chick aortic ring. Chick aortic rings were embedded in Matrigel and treated with different concentrations of ellagic acid stimulated by VEGF. The changes of sprout formations around various aorta samples were observed on the 3rd day (*left panel*; *a* 0 μM, *b* 2.5 μM, *c* 5 μM, *d* 10 μM). The index was defined as a percentage of the untreated control (*right panel*, values are represented as means ± SD, *n* = 6, *P < 0.05, **P < 0.01 versus untreated control). **b** The inhibitory effects of ellagic acid on sprout formation could be reversed after removal of ellagic acid from chick aortic ring. Aortic ring was initially fed with both VEGF and ellagic acid (10 μM) for 48 h (*left panel*), and then continually treated with VEGF after removal of ellagic acid for an additional 48 h (*right panel*). Images were representative of three independent experiments. **c** Ellagic acid could inhibit the microvessels formation on in vivo chick embryonic CAM model. Images were representative of three independent experiments (*left panel*). The index was defined as the mean number of visible microvessel branch with the defined area of drug-containing pellets on each CAM model (*right panel*, values are represented as means ± SD, *n* = 6, *P < 0.05, **P < 0.01 versus untreated control)

To mimic the in vivo angiogenesis situation, the organotypic assay in chick aortic arch model was then conducted by measurement of endothelial cell outgrowth [25]. It was found that sprouts around ellagic acid-treated rings were shorter and fewer cells migrated into the matrix, suggesting that ellagic acid could inhibit the sprout length and density after stimulation of VEGF (Fig. 3a). We further examined whether the inhibitory effects of ellagic acid were reversible. As shown in Fig. 3b, removal of ellagic acid contributed to sprout recurrence around the aortic ring, indicating that ellagic acid brought little toxic effects on normal vessel tissues. Besides, a different chick model system by in vivo chick embryonic CAM assay showed that the number of blood vessels gradually decreased with increasing concentrations of ellagic acid accompanied by VEGF stimulation (Fig. 3c). Overall, it was demonstrated that ellagic acid could inhibit sprouts formation from chicken aorta model and microvessels formation in CAM model.

### Ellagic acid attenuated VEGFR-2 tyrosine kinase activity and VEGFR-2 signaling pathway

**Fig. 4:**
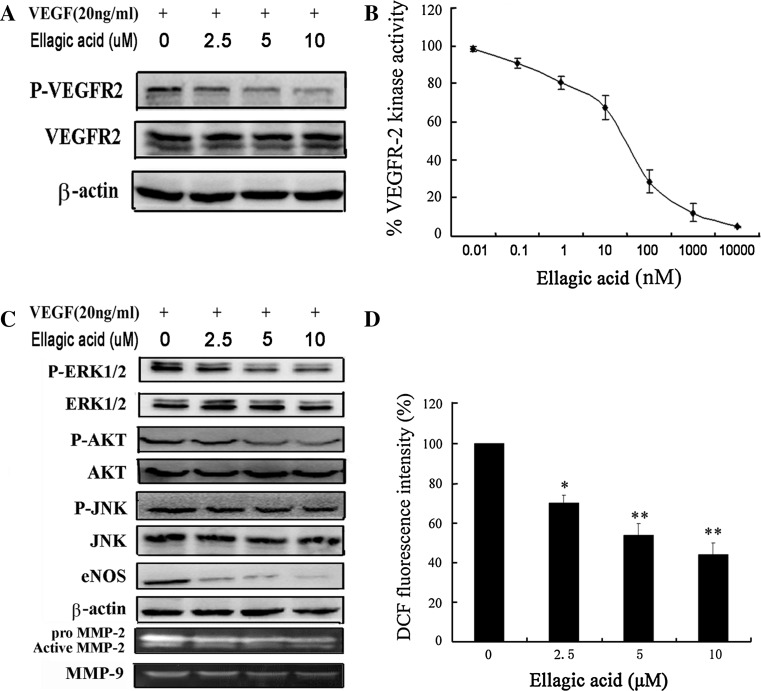
Ellagic acid attenuated VEGFR-2 tyrosine kinase activity and VEGFR-2 signaling pathway. **a** Ellagic acid reduced the phosphorylation of VEGFR-2 in VEGF-stimulated HUVECs. Endothelial cells were pre-cultured with ellagic acid at different concentrations for 24 h with the stimulation of VEGF (20 ng/ml). Expressions of VEGFR-2 and P-VEGFR2 were then examined by western blotting assay. It was found that P-VEGFR2 expression was markedly reduced, while the total level of VEGFR-2 was unaffected after the treatment of ellagic acid. **b** Ellagic acid suppressed VEGFR-2 kinase activity. VEGFR-2 and various concentrations of ellagic acid were incubated in kinase reaction buffer in 96-well plate coated with a poly-Glu-Tyr substrate. Phosphorylation of the substrate was monitored with a purified phosphotyrosine specific monocolonal antibody conjugated to horseradish peroxidase followed by chromogenic reaction with horseradish peroxidase substrate. Data are presented as a percentage of the control (values represent means ± SD, *n* = 3). **c** Ellagic acid inhibited VEGFR-2 downstream signaling molecules, including P-ERK/ERK, P-AKT/AKT, P-JNK/JNK, and e-NOS in a dose-dependant manner. Besides, the expression of MMP-2 and MMP-9 were also inhibited after ellagic acid administration. **d** The HUVECs intracellular ROS level was detected by DCFH-DA staining assay. The results showed that the intracellular ROS level was significantly decreased after ellagic acid treatment (values represent means ± SD, *n* = 6, *P < 0.05, **P < 0.01 versus untreated control)

Previous studies indicated that blockage of VEGFR-2 activity could significantly limit tumoral neo-angiogenesis process [26]. We first examined influences of ellagic acid on tyrosine phosphorylation of VEGFR-2 (the active form of VEGFR-2) stimulated by VEGF. The expression of P-VEGFR2 (Tyr 1175) and total VEGFR-2 were assessed by western blotting assay with their specific antibodies in the presence of VEGF. As shown in Fig. 4a, there was a significant reduction of P-VEGFR2 induced by ellagic acid, while the total levels of VEGFR-2 had little changes. We then investigated whether ellagic acid decreased P-VEGFR2 levels by inhibiting the kinase activity of VEGFR-2. Thus, ELISA-based tyrosine kinase assay was conducted to further examine the effects of ellagic acid on VEGF-stimulated P-VEGFR2. It was found that ellagic acid could dose dependently suppress kinase activity of VEGFR-2 with an IC_50_ of ~25.8 nM (Fig. 4b). In addition, previous studies supported that phosphorylation of VEGFR-2 could subsequently trigger multiple downstream signals that induced proliferation and differentiation activities of endothelial cells [27]. To better understand inhibitory effects of ellagic acid on VEGFR-2 and its downstream signaling, we next chose multiple essential downstream signaling molecules involved in VEGFR-2 activation for western blotting detection. Results showed that ellagic acid could evidently inhibit VEGF-stimulated eNOS expression and phosphorylation levels of ERK, AKT, and JNK, whereas the total expressions of the latter three were almost unaffected. In addition, both the MMP-9 and MMP-2 activities were suppressed after ellagic acid treatment (Fig. 4c). As ROS was also reported as a downstream signaling after VEGFR-2 activation [28], we also detect the ROS levels by DCFH-DA probe. The results showed that the intracellular ROS level was significantly reduced after ellagic acid administration (Fig. 4d). The above results revealed that ellagic acid inhibited in vitro angiogenesis by directly targeting VEGFR-2 on the surface of endothelial cells, and further suppressing VEGFR2-associated signaling pathways.

### Ellagic acid inhibited breast cancer growth and neoangiogenesis in vivo

**Fig. 5:**
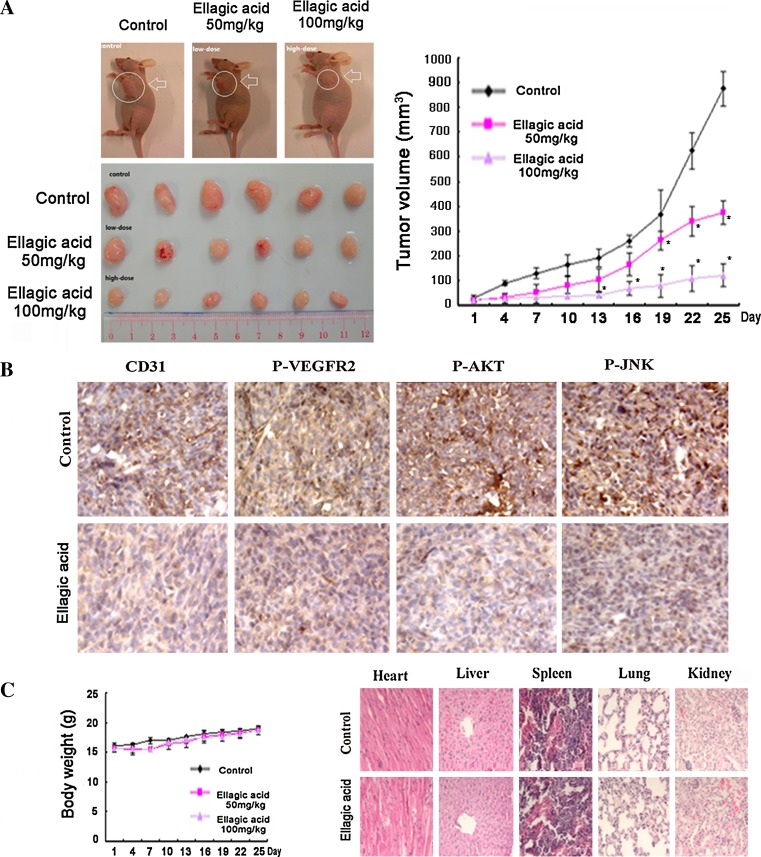
Ellagic acid inhibited growth and neoangiogenesis on MDA-MB-231 breast cancer xenografts. **a** Nude mice bearing MDA-MB-231 tumor were treated daily with the vehicle or ellagic acid at 50 or 100 mg/kg/day by intraperitoneal administration for 25 days. Representative mice with MDA-MB-231 xenografts and tumor masses were shown. Besides, it was found that treatment with ellagic acid obviously suppressed tumor volumes compared to the vehicle control group, indicating that ellagic acid could significantly inhibit the MBA-MD-231 tumor growth in vivo (values represent means ± SD, *n* = 6, *P < 0.05 versus vehicle group). **b** Tumor tissues were prepared for immunohistochemistry detection with specific antibodies against CD31, P-VEGFR2, P-AKT, and P-JNK. It was found that ellagic acid could obviously decrease tumoral microvessel density (MVD) indexed by CD31 in comparison with vehicle treatment. Meanwhile, ellagic acid treatment could obviously attenuate expressions of P-VEGFR2, P-Akt, and P-JNK, further demonstrating that ellagic acid played an important role in suppressing angiogenesis at least in part via VEGFR-2 signaling pathways in vivo. **c** Ellagic acid resulted in little toxicity effects in vivo. No significant differences of body weights were detected among all the groups (*upper panel*). Besides, it was found that ellagic acid did not cause apparent pathological abnormalities in the normal tissues including heart, lung, liver, spleen, and kidney, suggesting that there were no significant adverse effects of ellagic acid in vivo (*down panel*)

To investigate in vivo anti-angiogenesis effects of ellagic acid, breast cancer xenograft was built to evaluate whether ellagic acid could suppress tumor-induced angiogenesis in vivo*.* Previous studies indicated that MDA-MB-231 cell line was the first choice as pre-clinical breast cancer model owing to its high aggressive nature either in vitro or in vivo [29]*.* Thus, immunodeficient mice bearing MDA-MB-231 xenografts were treated daily with or without ellagic acid by intraperitoneal administration for 25 days. After mice were sacrificed, tumors and tissues were taken out for further analysis. Representative mice with MDA-MB-231 xenografts and tumor masses are shown in Fig. 5a. It was found that treatment with ellagic acid significantly led to suppression of MBA-MD-231 tumor volumes when compared with the control group treated with vehicle, suggesting that ellagic acid could inhibit tumor growth in vivo.

To further examine whether ellagic acid could suppress breast cancer growth by inhibiting angiogenesis, tumor tissues were stained with specific antibodies against CD31, P-VEGFR2 (Tyr 1175), P-AKT, and P-JNK in Fig. 5b. CD31 is a widely used endothelial marker for quantifying angiogenesis by calculating microvessel density (MVD) [30]. We found that vessels in vehicle-treated group were more numerous and stained more intensely compared with 100 mg/kg ellagic acid-treated group. In addition, ellagic acid treatment could obviously down-regulate expressions of P-VEGFR2, P-Akt, and P-JNK, further demonstrating that ellagic acid played an important role in suppressing angiogenesis at least partly through VEGFR-2 signaling pathways.

Meanwhile, no significant differences of body weights were detected among all the groups, indicating that ellagic acid administration brought little toxic effects on mice. Furthermore, normal tissue sections were embedded in paraffin and stained by hematoxylin and eosin (H&E) for toxicological analysis. No apparent pathological abnormalities were found in the normal tissues including heart, liver, spleen, lung, and kidney. Therefore, we could draw the conclusion that there were no significant adverse effects of ellagic acid in vivo, and the inhibitory effects of ellagic acid on cancer growth might not be due to systemic toxicity in mice. The result in Fig. 5c could provide an experimental basis for further study of ellagic acid in safe clinical application.

### Ellagic acid located at the ATP-binding sites of VEGFR-2 kinase domain

**Fig. 6:**
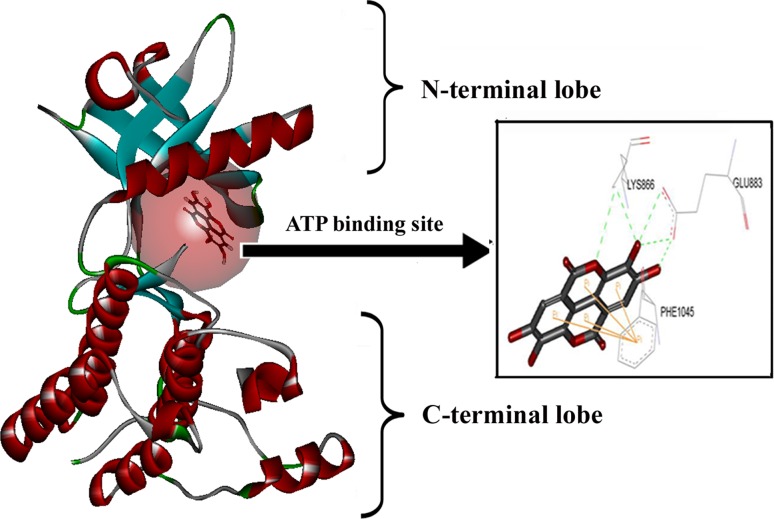
Ellagic acid interacted with the ATP-binding sites of VEGFR-2 kinase domain. The LigandFit algorithm in Discovery Studio 2.1 was applied to predict the binding mode of ellagic acid within ATP-binding domain of VEGFR-2 kinase. As anticipated, ellagic acid could stably bind to the ATP-binding pocket near the hinge region, which connected N- and C-lobes within VEGFR-2 catalytic domain. Specifically, ellagic acid formed hydrogen bonds with residues Lys866 and Glu883. Besides, ellagic acid could form strong π–π interactions with Phe1045

We next analyzed the binding pattern between ellagic acid and VEGFR-2 kinase domain to further understand how ellagic acid exerted anti-angiogenesis effects via VEGFR-2 and its signaling pathways. The ATP site within VEGFR-2 kinase domain was thus defined as the ligand-binding site based on the homology of 2-anilino-5-aryloxazole [31, 32]. As anticipated, ellagic acid could stably locate at the ATP-binding pocket near the hinge region, which connected N- and C-lobes within VEGFR-2 catalytic domain (Fig. 6). Three active residues, i.e., Lys866, Glu883, and Phe1045 at the ATP pocket were shown essential for the binding of ellagic acid with VEGFR-2. Particularly, ellagic acid could form hydrogen bonds with active residues Lys866 and Glu883, which were located at the hinge region and played an important role in identifying VEGFR-2 ligands. Besides, ellagic acid could form strong π–π interactions with Phe1045. The binding pattern of ellagic acid within VEGFR-2 provided structural insight for development of small natural inhibitors.

## Discussion

Extensive laboratory evidences supported that angiogenesis can be detected throughout the onset, growth, and metastasis in breast cancer [33]. Developing nontoxic anti-angiogenesis agents has thus become a promising strategy in breast cancer prevention and treatment. Ellagic acid is a dietary-derived polyphenol which has been widely reported to possess anti-cancer properties. In this study, we first focused on the inhibitory effects of ellagic acid on several steps of angiogenesis at the cellular level. It was found that ellagic acid could markedly inhibit angiogenesis-associated activities including proliferation, migration/invasion, and capillary formation on VEGF-stimulated endothelial cells. Besides, previous studies indicated that angiogenesis in vivo involved not only endothelial cells but also their surrounding cells [25]. Thus, chick aortic arch model was then used to mimic the in vivo process of angiogenesis. As anticipated, the sprouts formation stimulated by VEGF from ellagic acid-treated aorta was weak and scattered compared with the vehicle-treated groups. Nevertheless, the above in-vitro models lack the biological complexity of vascular system in vertebrate animals. Supporting evidences concerning in vivo anti-angiogenesis effects of ellagic acid then came from chick embryonic CAM model and MDA-MB-231 tumor xenograft model. Ellagic acid obviously inhibited blood vessels formation on CAM model and significantly suppressed tumor growth accompanied by a reduced MVD on tumor tissues in MDA-MB-231 xenograft model. Notably, we found that ellagic acid did not pose significant cytotoxicity to HUVECs at any tested concentrations based on LDH assay, indicating that the inhibitory effects of ellagic acid was not likely due to toxicity at the cellular level. In addition, vascular recurrence after ellagic acid removal on CAM models and no obvious toxic pathologic changes in normal tissues of mice xenograft model further demonstrated that ellagic acid could exert anti-angiogenesis effects with limited toxicity. These evidences might provide an experimental basis for further study of ellagic acid in safe clinical application.

We then verified the intrinsic mechanisms for the above anti-angiogenesis activities of ellagic acid. Inhibition of VEGFR-2 has been served as a prosperous strategy for angiogenesis therapeutic intervention [34]. By functionally coupling with VEGF, VEGFR-2 would undergo autophosphorylation mainly at Tyr1175 sites within its intracellular kinase domain and then initiate a series of downstream signal transductions to endothelial cells [35]. Our findings revealed that ellagic acid could suppress Tyr1175 phosphorylation of VEGFR-2 stimulated by VEGF as well as VEGFR-2 tyrosine kinase activity. Multiple VEGFR-2 downstream signaling mediators such as ERK, AKT, JNK, and eNOS were also involved in regulation of endothelial cells survival and proliferation. According to previous supporting evidences [36, 37], P42/44 ERK activation attributes to an increased proliferation on endothelial cells; AKT is responsible for the survival of endothelial cell; JNK activation induces proliferation and migration on endothelial cells by promoting nuclear activation of c-Jun; and eNOS plays a key role in vascular permeability and migration activities on endothelial cells. Our results demonstrated that multiple MAPK signaling mediators including p-ERK, p-AKT, p-JNK, and eNOS were inhibited by ellagic acid. In addition, it was also found that ellagic acid dose dependently inhibited MMPs activity, confirming the previous findings that decreased MMPs activity might be also responsible for interfering with the binding of VEGF to VEGFR-2, and therefore inhibiting the neo-angiogenesis process [38]. Furthermore, ROS was reported as a downstream signaling of VEGFR-2 and served as a survival mediator in supporting endothelial cells proliferation [28]. Our results showed that the ROS level was significantly decreased after ellagic acid administration, which might be a consequence event of decreased VEGFR-2 activity. All these results showed that ellagic acid treatment suppressed the VEGFR-2 pathways. Meanwhile, the findings in vitro were also consistent with our in vivo results, indicating that the reduced MVD value may owe to the suppression activities of ellagic acid on the expression of phosphorylated VEGFR-2 and its downstream signaling molecules.

To thoroughly understand how ellagic acid interacted with VEGFR-2 to exert anti-angiogenesis effects, we further examined the structure-based interaction between ellagic acid and VEGFR-2 in silico. The identification of VEGF with VEGFR-2 is tightly associated with the second/third immunoglobulin-like regions within the VEGFR-2 extracellular domain. Besides, the downstream structure from the fourth to seventh immunoglobulin-like domains in VEGFR-2 plays a major role in dimerization and activation [39]. Disappointedly, it has been shown that the overall structure of VEGFR-2 extracellular domain is excessively flexible and change too much before and after binding with VEGF. Therefore, this dynamic nature prevented extracellular domain to be a good target for small molecular inhibitors.

As mentioned above, dimerization within the extracellular domain of VEGFR-2 could induce the autophosphorylation on numerous tyrosine residues within its intracellular domain. The phosphorylation is an ATP-consuming process. The ATP-binding region lies between N-terminal lobe and C-terminal lobe within VEGFR-2 catalytic domain. Many kinase inhibitors could exert their inhibitory effects through purely or partially competing against the adenosine triphosphate (ATP) and subsequently suppressing the receptor autophosphorylation. They were acting as ATP minetics that bound to this site and competed with cellular ATP [9, 11–13]. In this study, ellagic acid could stably locate at the ATP-binding pocket near the hinge region. At least 16 active residues existed within the catalytic unit of VEGFR-2, including Val846, Phe1045, Cys1043, Leu1033, Asn1031, Arg1030, Asn921, Gly920, Lys918, Cys917, Phe916, Val914, Lys866, Ala864, Gly841, Glu883, and Leu838. Only three active residues, i.e., Lys866, Glu883, and Phe1045 at the ATP pocket were essential for the stable conformation of VEGFR-2/ellagic acid complex. The binding mode of ellagic acid with VEGFR-2 differed considerably from that of 2-anilio-5-aryoxazole (1Y6B) [31], although they shared similar aromatic interaction with the same residue Phe1045. In addition to the strong π–π interactions with Phe1045, ellagic acid could also form hydrogen bonds mainly with two residues, i.e., Glu883 and Lys866. All the unique binding modes largely promoted the conformational stability of the ellagic acid/VEGFR-2 complex.

Overall, our study indicated that ellagic acid at non-toxic dosages exerted potent anti-angiogenesis activities via specifically targeting VEGFR-2 and its signaling pathway in breast cancer. As a natural inhibitor against VEGFR-2 with limited toxicity, ellagic acid is a promising candidate for development of anti-angiogenesis agents.
